# Changes of serum inflammatory markers and immune function in children with bronchial asthma complicated with mycoplasma pneumoniae infection before and after treatment and their clinical significance

**DOI:** 10.12669/pjms.41.5.11067

**Published:** 2025-05

**Authors:** Xiao-jun Shi, Hui-min Tian, Cai-xia Li, Jin-kai Wei

**Affiliations:** 1 Xiao-jun Shi Department of Pediatrics, Beijing JingDu Children’s Hospital, Changping District, 102200 Beijng, China; 2 Hui-min Tian Department of Pediatrics, Baoding First Central Hospital, Baoding 071000, Hebei, China; 3 Cai-xia Li Department of Pediatrics, Baoding First Central Hospital, Baoding 071000, Hebei, China; 4 Jin-kai Wei Department of Pediatrics, Baoding First Central Hospital, Baoding 071000, Hebei, China

**Keywords:** Bronchial asthma, Inflammatory marker, Immune function, Mycoplasma pneumoniae, Treatment

## Abstract

**Objective:**

To evaluate the changes of serum inflammatory markers and immune function in children with bronchial asthma complicated with Mycoplasma pneumoniae infection before and after treatment.

**Methods:**

This was a retrospective study. Thirty children with bronchial asthma complicated with mycoplasma pneumoniae infection were selected as the experimental group and 30 healthy children as the control group at Baoding First Central Hospital from December 2022 to December 2023. The levels of inflammatory markers interleukin-2(IL-2), interleukin-4(IL-4), Serum Amyloid A(SAA), Procalcitonin(PCT), immune markers immunoglobulin A(IgA), immunoglobulin G(IgG), immunoglobulin M(IgM) and CD4^+^ were detected before and after treatment, and the changes were compared with those of the control group.

**Results:**

Compared with the control group before treatment, the levels of immune markers, inflammatory markers IL-2, IL-4 and SAA in the experimental group were significantly different (p < 0.05). However, there was no significant difference between the two groups in PCT (p > 0.05) level. In the experimental group, except PCT, the levels of other inflammatory markers and immune markers were significantly improved after treatment, and the difference was statistically significant (P < 0.05).

**Conclusion:**

Inflammatory markers and immune function of children with bronchial asthma complicated with Mycoplasma pneumoniae infection were abnormal to a certain extent, and the improvement was obvious after treatment. The result may have certain reference value for diagnosing the disease and determining its treatment effects.

## INTRODUCTION

Bronchial asthma is a common chronic respiratory tract disease. The causes are complex and may be related to chronic respiratory tract infections, inflammation, allergy and other factors, which eventually lead to airway hyperresponsiveness.^1^ Tracheal spasm often occurs once the patient is stimulated by dust, lampblack and allergic substances in the external environment^2^, causing great harm. Common pathogens of respiratory tract infections include Mycoplasma pneumoniae, Chlamydia, Adenovirus and Respiratory Syncytial Virus.^3^ Mycoplasma pneumoniae is adsorbed on mucosal epithelial cells of the respiratory tract, which leads to respiratory mucosal epithelium damage and dysfunction through oxidative stress reaction, causing respiratory mucosal epithelium clearance dysfunction and increased reactivity.^4^

In recent years, there have been more in-depth studies on the interaction between bronchial asthma and Mycoplasma pneumonia. Meyer et al.^5^ believe that it is related to immune response, that is, immune response caused by Mycoplasma pneumoniae infection induces or aggravates bronchial asthma. Cruz et al.^6^ believe that, after infecting the body, Mycoplasma can cause an imbalance in inflammatory markers such as interleukin-4(IL-4), interleukin-2(IL-2), Serum Amyloid A(SAA), in the airway and further lead to hyperresponsiveness in the airway. In addition, Mycoplasma can activate the coagulation system of the human body and cause corresponding dysfunction in the system.^7^ At present, there are few reports on the changes of immune conditions, coagulation functions and inflammatory markers in children with bronchial asthma complicated with Mycoplasma pneumonia before and after treatment. We have selected thirty children with the disease to analyze the changes before and after treatment, in order to provide certain objective basis for the early diagnosis of the disease and the judgment of treatment effects.

## METHODS

This was a retrospective study. Thirty children with bronchial asthma complicated with mycoplasma pneumoniae infection were selected as the experimental group and 30 healthy children as the control group at Baoding First Central Hospital from December 2022 to December 2023. Thirty children patients were selected in the experiment group, including 18 males and 12 females, aged 7-13 years old, with an average at 6.7±1.5 years old.

### Ethical approval:

The study was approved by the Institutional Ethics Committee of Baoding First Central Hospital(No.: [2020] 068; November 4,2020), and written informed consent was obtained from guardians of all the participants.

### The diagnostic criteria of bronchial asthma^8^

- They have developed multiple contact allergen symptoms such as repeated asthma and shortness of breath.
- Scattered or diffuse wheezing of both lungs can be heard at the respiratory phase during the asthma attack, with a prolonged respiratory phase.
- Symptoms and signs were effectively relieved by anti-asthma treatment or relieved spontaneously;
- The possibilities of other disease causing asthma, cough, shortness of breath and chest tightness were excluded.
- Aged 7-13 years old.
- Mycoplasma hemopneumoniae antibody MP-IgM was positive (antibody titer ≥ 1:80).
- The family members of the children conformed to their participation in the experiment and signed an informed consent form.

### Criteria for the exclusion of cases:

- Bronchial asthma complicated with other pathogen infections, such as bacterial infection and fungal infection.
- The cases suffered from immune diseases or chronic inflammation that interfered with the research;
- The cases were orally taking immunosuppressants or hormone drugs.

At the same time, 30 healthy children were selected as the control group, including 15 males and 15 females. The age ranged from four to twelve years old, averaging at 7.5 ± 1.4 years old. There was no significant difference between the two groups in general data (p > 0.05), which made both comparable. The experimental group was given symptomatic treatment, such as spasmolysis, expectorant, cough relieving, oxygen inhalation, according to the children’s condition. At the same time, azithromycin granules were applied orally at a dose of 10mg/kg on the first day, with the maximum amount not exceeding 0.5 g per day. From Day two to Day five, the dose of 5mg/kg was taken orally at a single time, with the maximum amount not exceeding 0.25 g per day. The course of treatment was five days. Budesonide formoterol powder inhalant (budesonide/formoterol: 80/2.25 μg), were taken twice a day, with one inhalation each time. The course of treatment was seven days.

### Observation Indexes:

Before treatment and after treatment (seven days after treatment), fasting blood was taken from the children in the experiment group in the morning to detect inflammatory markers IL-4, CRP, SAA, PCT (ELISA method) and immune markers IgA, IgG, CD4 +, IgM (immunoturbidimetry used on Hitachi 7600 automatic biochemical analyzer). At the same time, 30 healthy children in the control group were taken fasting blood in the morning and the above indexes were detected as controls.

### Statistical Analysis:

All the data were analyzed using SPSS 23.0, and the measurement data were expressed as (± S). The data analysis between the experimental group and the control group was conducted by two groups of independent samples t-test. The comparative analysis of each index before and after treatment in the experimental group was carried out by paired t-test. A result with P < 0.05 was considered statistically significant.

## RESULTS

Compared with the control group before treatment (Table-I), the experimental group showed significantly different (P < 0.05) levels of immune markers, inflammatory markers IL-2, IL-4 and SAA. However, there was no significant difference in PCT between the two groups (p > 0.05). After treatment, the level of inflammatory markers in the experimental group was reexamined, showing obvious improvements compared with that before treatment. Except that there was no significant difference in PCT after treatment, the difference of other markers was statistically significant (p < 0.05) (Table-II). The level of immune markers in the experimental group improved significantly after treatment, and the difference of each index was statistically significant (P < 0.05) (Table-III).

**Table-I:** Comparative Analysis of the Indexes between the Experimental Group and the Control Group before Treatment (*χ̅±S*) n=30.

Indexes	Inflammatory markers(ng/L mg/L)				Immune markers(Ig= g/L)*			
Category	IL-4*	IL-2*	PCT^Δ^	SAA*	CD4 +	IgG	IgA	IgM
Experimental group	18.16±2.33	240.31±30.12	0.22±0.05	72.25±17.28	31.17±6.07	12.05±3.13	1.32±0.32	2.43±0.87
Comparative group	5.04±1.37	332.27±25.39	0.20±0.07	3.82±1.13	42.03±5.74	6.91±2.47	1.17±0.13	1.35±0.46
*t*	25.97	12.79	1.27	24.99	7.12	4.47	2.38	6.01
*p*	0.00	0.00	0.21	0.00	0.00	0.00	0.02	0.00

*Note:* IL4, IL2, PCT=ng/L, SAA=mg/L; PT, APTT, TT=S; FIB=g/L

* p<0.05, Δp>0.05

**Table-II:** Comparative analysis of the indexes before and after treatment in the experimental group (*χ̅±S*) n=30.

Indexes	Before Treatment	After Treatment	t	p
IL-4(ng/L)*	18.16±2.33	7.32±2.18	18.61	0.00
IL-2(ng/L)*	240.31±30.12	309.79±29.83	9.06	0.00
PCT(ng/L)	0.22±0.05	0.23±0.12	0.42	0.67
SAA(mg/L)*	72.25±17.28	3.71±1.06	25.04	0.00

* p<0.05

**Table-III:** Comparative Analysis of Immune Markers in the Experimental Group before and after Treatment (*χ̅±S*) n=30.

Indexes	Before Treatment	After Treatment	t	p
IgG(g/L)	12.05±3.13	6.72±2.39	7.59	0.01
IgA(g/L)	1.32±0.32	1.10±0.35	2.54	0.02
IgM(g/L)	2.43±0.87	1.41±0.72	4.89	0.00
CD4 +	31.17±6.07	43.12±5.47	8.41	0.00

p<0.05

## DISCUSSIONS

The contents of serum IgG, IgA and IgM in 30 children in the experimental group were higher than those in the control group, and the difference between the two groups was statistically significant(p< 0.05). The results of CD4 + were similar to those reported in the literature^9^, suggesting that the immune function of children with bronchial asthma complicated with Mycoplasma infection was somewhat disturbed or out of balance. Studies have proved^10^ that Mycoplasma pneumoniae infection can lead to the immune imbalance of body cells and humoral fluids, raising the contents of immunoglobulin IgG, IgA and IgM to a level higher than normal, while the contents of CD3 + and CD4 + are reduced. Imbalance of the immune status can aggravate inflammatory reaction and cause damage to airway structure and function.^9^ The essence of the onset of bronchial asthma is airway inflammation.^11^ Pathogenic microorganisms can stimulate the body to produce antibodies and produce a large number of inflammatory markers and mediators through immune response, thus promoting the occurrence of airway hyperresponsiveness.^12^

IL-2 and IL-4 are two common inflammatory markers, which play a very important role in the occurrence and development of inflammation. The former is mediated by T lymphocyte helper cell type 1 (Th1), while the latter is mediated by T lymphocyte helper cell Type-2 (Th2)^13^, which participates in cellular immunity and humoral immunity respectively. In this study, the level of IL-2 in the experimental group was significantly lower than that in the control group before treatment, and the level of IL-4 was significantly higher than that in the control group, with significant differences (p=0.00, p=0.00). IL-4 can stimulate B lymphocyte proliferation, inhibit cellular immune function, enhance humoral immune activity, aggravate airway edema, tracheal spasm and stenosis, and aggravate bronchial asthma symptoms.

Serum amyloid A (SAA) is secreted by hepatocytes.^14^ It will increase rapidly in the short- and medium-term in infectious diseases, and the increase is more obvious in Mycoplasma infection. SAA has high specificity for mycoplasma pneumoniae infection in children, and can be used as a detection index for auxiliary diagnosis of early Mycoplasma pneumoniae infection in children.^15^ SAA is an acute solid protein, which increases rapidly within six hours after infection. However, after the infection is subdued, it quickly decreases to normal level. The results of this study suggested that SAA was significantly higher than that of the control group before treatment (p=0.00) and basically decreased to the normal level after reexamination, with significant difference before and after treatment (p=0. 00). Therefore, SAA can play a role in not only the diagnosis of diseases but also the judgment of treatment effects. PCT mainly reflected bacterial infection and had no obvious change in Mycoplasma infection. Therefore, it was not helpful for the diagnosis and treatment effect judgment of the disease. Bronchial asthma complicated with Mycoplasma pneumoniae infection mostly adopted symptomatic treatment combined with antibiotic treatment.

Pathogenic microorganism infection, immunity responses, neuroendocrine are related to the pathogenesis of bronchial asthma, of which Mycoplasma infection is an important factor for its occurrence and development.^16^ Mycoplasma pneumoniae (MP) parasitizes on the surface of respiratory tract mucosa after infecting human body, releasing hydrogen peroxide, nuclease and other substances that cause necrosis of respiratory tract epithelial cells, pathological changes, infiltration of inflammatory cells such as lymphocytes, monocytes, macrophages and the like^17^; It also causes thickening of respiratory tract mucosa, stenosis of the lumen, sensitization and increased sensitivity of submucosal nerve fiber endings, inducing or aggravating asthma symptoms such as wheezing and chest tightness.

Symptomatic treatment includes spasmolysis, antiasthma, expectorant, antipyretic and liquid supplement. Azithromycin achieves antibacterial effect by combining with ribosomal 50S subunit in bacterial cells, hindering the process of bacterial transpeptide and inhibiting the synthesis of RNA-dependent proteins.^18^,^19^ However, due to structural changes, azithromycin has a wider antibacterial spectrum than erythromycin and can inhibit a variety of Gram-positive cocci, Mycoplasma and chlamydia. Budesonide formoterol powder inhalant is a composite preparation. Budesonide has good anti-inflammatory effect, can inhibit the aggregation of inflammatory markers and the release of inflammatory mediators, and reduces airway hyperreactivity^20^, therefore, it can not only quickly relieve symptoms, but also block the further development of the disease. Formoterol is a two receptor agonist, which can relax bronchial smooth muscle, relieve ischemia^21^, improve cilia function and enhance local resistance after inhalation. Small dosage of budesonide/formoterol inhalant (budesonide/formoterol: 80/2.25g) is safe and effective for children aged 6-12 years old and can be inhaled twice a day.^22^

This study confirmed that the serum inflammatory markers and immune function of the children were improved to some extent after treatment, and the clinical symptoms of the children were relieved. Therefore, it also indicated that inflammatory markers and immune markers played a positive role in evaluating the treatment effect of the disease. However, the changes of PCT were not obvious before and after treatment, which may be attributed to the fact that PCT changes obviously in bacterial infection, with no obvious impact on other microbial infections such as Mycoplasma infection.

### Limitations:

The sample size was relatively small, so there is no detailed classification of bronchial asthma complicated with Mycoplasma infection. Further research with increased sample size and detailed classifications on Mycoplasma infection is necessary. The study collected data in a relatively short period of time after treatment. After about one week of treatment, reexamination and data collection were carried out for comparison. Therefore, although some indexes have improved obviously, they have not yet reached the normal level. If reexamination is carried out after a longer treatment time, they may continue to improve until the normal level is reached.

## CONCLUSIONS

Children with bronchial asthma complicated with Mycoplasma infection may have certain abnormalities in immune function compared with normal population, which further lead to abnormal levels of inflammatory markers. After symptomatic treatment such as anti-inflammatory, antiasthmatic and expectorant treatment, all indexes except PCT were obviously improved, suggesting that indexes such as immunity and inflammatory factors have certain reference value for not only the diagnosis of diseases, but also the judgment of the treatment effect and prognosis.

### Authors’ Contributions:

**XJS** and **HMT:** Designed this study and prepared this manuscript, and is responsible and accountable for the accuracy or integrity of the work.

**CXL:** Literature search, Collected and analyzed clinical data.

**JKW:** Literature sarch, critical review and revised this manuscript.

All autors have read, approvred the final version and are accountable for the integrity of the study.
